# Early transmural response assessed by intestinal ultrasound predicts long-term clinical remission in patients with IBD: Results from the TRUST BEYOND study

**DOI:** 10.1093/ibd/izag074

**Published:** 2026-05-19

**Authors:** Torsten Kucharzik, Ulf Helwig, Frank Seibold, Luc Biedermann, Christoph Högenauer, Imma Fischer, Leonie Hammer, Stefan Rath, Christian Maaser

**Affiliations:** Department of Internal Medicine and Gastroenterology, University Teaching Hospital Lüneburg, Lüneburg, Germany; Gastroenterology Practice, Oldenburg, Germany; University of Kiel, Kiel, Germany; Crohn Colitis Centre, Bern, Switzerland; University Clinic Zurich, Zurich, Switzerland; Division of Gastroenterology and Hepatology, Department of Internal Medicine, Medical University of Graz, Graz, Austria; Biostatistics Tübingen, Tübingen, Germany; Medical Department, AbbVie Germany GmbH & Co. KG, Wiesbaden, Germany; Medical Department, AbbVie Germany GmbH & Co. KG, Wiesbaden, Germany; Center of Physiology, Pathophysiology and Biophysics, Paracelsus Medical University, Nuremberg, 90419, Germany; Outpatients Department Gastroenterology, University Teaching Hospital Lüneburg, Lüneburg, Germany

**Keywords:** inflammatory bowel diseases, ultrasound, predictors

## Abstract

**Background:**

The study sought to prospectively examine clinical, biochemical, and transmural outcomes. Transmural outcomes are assessed by ultrasound after 1 year in patients with inflammatory bowel disease (IBD) treated in line with standard of care.

**Methods:**

The TRUST BEYOND study is a prospective, multicenter, noninterventional study at 49 sites in Germany, Austria, and Switzerland and includes 282 patients with IBD who have active disease at baseline. For this analysis, 163 patients with IBD (74 with Crohn’s disease, 89 with ulcerative colitis) were followed up for 1 year. At baseline and at every 3 months, clinical, biochemical, and ultrasound parameters, such as bowel wall thickness and color Doppler sonography, were assessed.

**Results:**

Patients with IBD demonstrated vast improvements in clinical, biochemical, and ultrasound assessments as early as week 12. Rates of transmural response and healing progressively increased over the study period to up to 77.5% and 39.3%, respectively, in patients with ulcerative colitis and to 66.2% and 27.0%, respectively, in patients with Crohn’s disease. Patients with early transmural response achieved numerically higher rates of various outcomes at week 52 than patients without early transmural response. In a multivariate analysis, transmural response at week 12 predicted clinical remission at week 52 (odds ratio, 4.3; 95% confidence interval, 2.45-7.52; *P* < .001).

**Conclusions:**

These results suggest that intestinal ultrasound is a suitable noninvasive modality to monitor patients with IBD in routine care. Transmural endpoints can be achieved in patients with IBD treated with standard of care and can predict outcomes after 1 year.

## Graphical Abstract

**Graphical Abstract:**
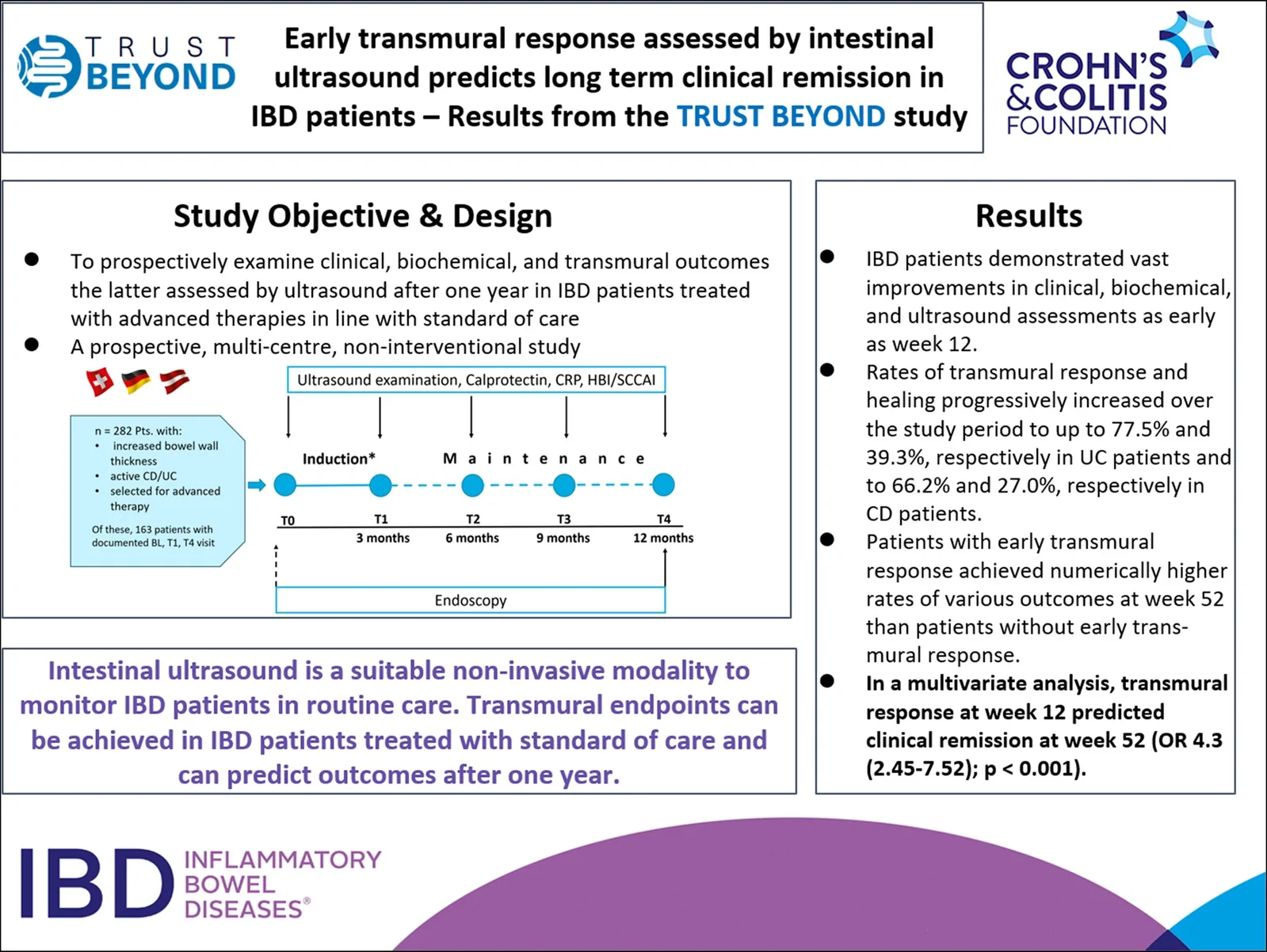

Key messages **What is already known:** Intestinal ultrasound (IUS) is a noninvasive, low-cost alternative to monitor response to therapy in patients with Crohn’s disease (CD) and those with ulcerative colitis (UC), and its use is increasing worldwide.

**What is new here:** IUS improvements after therapy induction predicted positive patient outcomes after 1 year. Response kinetics differ between patients with CD and those with UC.

**How can this study help patient care:** IUS is a suitable diagnostic modality for monitoring patients with CD and those with UC. Transmural response is achievable early after initiation with advanced therapy and might become a novel treatment target in inflammatory bowel disease management.

## Introduction

Inflammatory bowel diseases (IBDs), including Crohn’s disease (CD) and ulcerative colitis (UC), are of a relapsing-remitting nature with episodes of remission interspersed by intervals of active inflammation.

Since the STRIDE (Selecting Therapeutic Targets in Inflammatory Bowel Disease) initiative firstly proposed a shift in patient management in IBD, the IBD community has witnessed an evolution from a mere symptom-focused treatment strategy to a treat-to-target (T2T) approach including tight control.^1^

Mucosal healing is one of the objective treatment targets defined by STRIDE-II. Mucosal healing, usually defined as the absence of ulcerations during endoscopy, is associated with improved long-term outcomes and patients achieving mucosal healing are more likely to be in steroid-free remission and are known to have reduced rates of hospitalization and surgery.^2–4^

Recently, transmural healing—potentially representing a deeper level of healing—has moved into the spotlight as a novel therapeutic aim in IBDs. Having updated its recommendations of 2015, the STRIDE initiative considers transmural healing to be an adjunctive measure in CD.^1^ Compared with mucosal healing, transmural healing may provide a more accurate assessment of both transmural and peri-intestinal disease burden in CD and UC.^5–9^ This is a key issue not only in CD, in which intestinal inflammation affects all layers of the bowel, but also in UC, in which submucosal oedema, cellular infiltrate, and fibrosis are present as well.^10^^,^^11^ Moreover, Castiglione et al^12^ showed that patients with CD who achieved transmural healing after anti-tumor necrosis factor (TNF) therapy had significantly better outcomes than patients who experienced mucosal healing or no healing. The relevance of transmural healing on long-term outcomes has now been confirmed by several other groups.^13–16^ While to some degree, mucosal healing and transmural healing exhibit a correlation, the latter probably reflects a more comprehensive and pronounced level of healing.^17^

The diagnostic armamentarium to assess transmural healing encompasses cross-sectional imaging modalities including magnetic resonance elastography, computed tomography, and intestinal ultrasound (IUS).^18^

IUS has gained increasing relevance as an objective, noninvasive, patient-centric, radiation-sparing modality to diagnose and monitor patients with CD and UC.^18^ Furthermore, intestinal inflammation and treatment response by IUS can be thoroughly evaluated and visualized in real time. This feature provides the opportunity to directly interact with the patient and to substantially support the patient in understanding the disease which might increase patient compliance.^19^ According to Goodsall et al,^19^ patients prefer IUS over other diagnostic modalities including endoscopy. IUS is widely available, inexpensive, and well tolerated by patients, as it does not require any bowel preparation in contrast to endoscopy.^20^ In contrast to endoscopy, IUS has the ability to detect transmural inflammation by visualizing different bowel wall layers and identifying extramural abnormalities.

IUS provides the opportunity to repeatedly evaluate short- and long-term response to therapy to quickly detect relapses and, together with the introduction of new therapeutic modes of actions, to modify treatment decisions accordingly. Its use is recommended by the international European Crohn’s and Colitis Organization guidelines with landmark studies such as the TRUST and TRUST&UC study demonstrating its applicability and monitoring options in routine practice in IBD.^5^^,^^6^ In line with this, IUS is increasingly used to assess clinical efficacy in interventional trials such as the STARDUST trial (NCT03107793) with ustekinumab or the AFFIRM study with risankizumab (NCT06063967).^21^

The most relevant IUS parameters in IBD are bowel wall thickness (BWT) and vascularity determined by color Doppler sonography (CDS). According to the recently published consensus statement for transmural healing defined by IUS, treatment response is most commonly defined as a reduction in BWT and CDS.^22^ Although there is less literature on IUS and transmural healing in UC compared with CD, most experts agree that the evaluation criteria are similar for both conditions.^22^^,^^23^

Given the strong evidence for using IUS to assess transmural inflammation and early therapy response, the next step is to evaluate how well transmural healing by IUS predicts long-term treatment outcomes.

Numerous studies have paved the way for IUS, not only as a monitoring tool, but also as a predictive modality in IBD. With this regard, several IUS parameters have been explored as predictive markers.^17^^,^^24–26^

Most of these studies, however, were conducted as single-center studies, and they had a small sample size and only investigated either endoscopic or clinical outcomes. Prospective large-scale studies are required to confirm the predictive value of transmural healing assessed by IUS before IUS can be established as a treatment target for patients with both CD and UC in a T2T setting. Here, we present data from the prospective, multicenter, multicountry TRUST BEYOND study demonstrating that early transmural response has, to some degree, a predictive value for the clinical and sonographic outcome after 1 year.

## Methods

### Patients and study design

The TRUST BEYOND study is a multicenter, prospective, noninterventional study that was conducted in Germany, Austria, and Switzerland. The TRUST BEYOND study focused on patients with clinically active CD or UC across 49 study sites, representing various levels of care, including general practices and both in- and outpatient departments. The first patient was enrolled in January 2019. Adult patients (≥18 years of age) with CD who had a proven diagnosis of ileocolonic and/or colonic CD as well as adult patients with UC who had a proven diagnosis of proctosigmoiditis, left-sided colitis, or pancolitis that were selected for therapy with biologicals or Janus kinase (JAK) inhibitors prior to study inclusion were consecutively enrolled. At baseline, patients had to have active disease (CD: Harvey-Bradshaw Index [HBI] ≥ 7; UC: Simple Clinical Colitis Activity Index [SCCAI] ≥ 5) and an increased BWT (>4 mm for sigmoid colon, >3 mm for other segments). Patients were treated with advanced therapies according to the standard of care at the discretion of the individual physician. At the time of study start, the approved therapies included anti-TNF inhibitors (adalimumab, infliximab, golimumab), anti-integrin inhibitor (vedolizumab), anti-interleukin-12/23 inhibitor (ustekinumab), and JAK inhibitor (tofacitinib). All patients gave written informed consent prior to study inclusion.

At baseline, demographic, disease history–related data, sonographic, and laboratory (fecal calprotectin [FCAL] [by both home test and site test] and C-reactive protein [CRP]) and clinical parameters (HBI and SCCAI) were collected. Endoscopic data (per Simple Endoscopic Score for CD [SES-CD] and Mayo Score for UC) was documented if available. Endoscopic remission was defined as a Crohn’s Disease Endoscopic Index of Severity of <4 without deep ulceration for CD and a Mayo endoscopic subscore of ≤1 for UC. During the study, up to 4 visits were scheduled: baseline, after 3 months, after 6 months, after 9 months, and after 12 months (results of the STARDUST CD substudy were not published at the time of study start, and assessment time points were based on the TRUST study). An optional visit could be done after 24 months. At each follow-up visit, sonographic, clinical, and laboratory data were documented as well as changes in IUS-specific medications and endoscopic data if both were available. The primary endpoint of the study was the proportion of patients with clinical and steroid-free remission after 12 months following transmural response after 3 months. Clinical remission was defined as HBI <5 for patients with CD and SCCAI ≤2 for patients with UC. To reach steroid-free remission, patients had to be steroid-free and in clinical remission at the time of the visit.

### IUS examination

At each study visit, all bowel segments were examined by IUS. Prior to study start, IUS parameters and standards of the IUS assessment were discussed at an investigator meeting at which the investigators underwent training with simulators to ensure a consistent investigation procedure. The evaluated segments encompassed the terminal ileum (in patients with CD only), ascending colon, transverse colon, descending colon, and sigmoid colon. In total, the following IUS parameters were assessed: BWT, CDS, loss of stratification, loss of haustration, presence of strictures, fistulas, stenoses, abscesses, presence of inflammatory fat, and presence of mesenteric lymphadenopathies. Patients underwent the IUS assessment without special preparation. BWT was documented in millimeters for each individual segment. To minimize limitations when measuring in 1 scan plane, BWT was calculated as the mean of 4 measurements: 2 each in the cross-sectional and longitudinal scan planes. Vascularity was assessed by CDS, applying the modified Limberg score for patients with CD. For patients with UC, due to the lack of a comparable scoring system, a simple dichotomous analysis was conducted. Investigators reported either an increased or a normal CDS as done previously in the TRUST&UC study. The velocity range for the CDS was set to 57 cm/s due to dimensions of the gut vessels and small blood flow.

Transmural response was defined as at least 25% reduction of BWT and/or normalization of the BWT (≤3 mm) in the most affected segment (segment with the highest BWT). Transmural remission was defined as normalization of BWT and normalization of CDS (CD: Limberg score ≤1; UC: no increased signal). All other IUS parameters were analyzed in a dichotomous way (present or absent).

### Statistical analysis

For descriptive statistics, patients were analyzed both as an IBD group and as either a CD or UC subgroup, based on their IBD entity. Median (interquartile range [IQR]) was used to describe quantitative variables, and absolute and relative frequencies were used for discrete variables. All tests in the analysis were 2-sided, assuming a 5% error probability (α = 0.05). For exploratory uni- and multivariate analyses, parameters were depicted with odds ratio (95% confidence interval) and *P* values. For the primary endpoint (clinical remission at week 52), we applied a nonresponder imputation approach. Other variables (such as lab values or ultrasound findings) were not imputed but left as missing; analyses for these were performed on an available-case basis. The number of documented values for each variable is shown in Tables 1 and 2.

**Table 1:** Patient demographics at baseline of the ITT population.

	**IBD (n** = **163)**	**CD (n** = **74)**	**UC (n** = **89)**
**Female**	44.2 (72)	52.7 (39)	37.1 (33)
**Age, y**	34.6 (27.8-51.9)	30.9 (25.7-39.9)	40.8 (30.6-55.7)
**Time since diagnosis, y**	5.21 (1.74-10.69) (n = 156)	3.96 (1.36-10.31) (n = 70)	5.76 (2.49-11.53) (n = 86)
**Disease extension on last endoscopy**	See IBD subtype	L1 (ileum): 31.1 (23)	Proctosigmoiditis: 14.6 (13)
		L2 (colonic): 24.3 (18)	Left-sided colitis: 36.0 (32)
		L3 (ileal-colonic): 37.8 (28)	Pancolitis: 41.6 (37)
		L4 (upper GI tract): 4.1 (3)	Not assessable: 7.9 (7)
		Not assessable: 2.7 (2)	
**Clinical disease activity score**			
** HBI**	10.0 (8.0-12.0) (n = 74)	10.0 (8.0-12.0) (n = 74)	—
** SCCAI**	8.0 (7.0-10.0) (n = 89)	—	8.0 (7.0-10.0) (n = 89)
**FCAL, µg/g**	800 (381-1681) (n = 96)	665 (386-2024) (n = 42)	800 (264-1427) (n = 54)
**CRP, mg/L**	11.7 (4.60-38.0) (n = 148)	16.9 (5.3-37.5) (n = 69)	10.0 (3.0-38.0) (n = 79)
**Endoscopic score**	See IBD subtype	SES-CD: 11.00 (8.00-16.00) (n = 29)	MES 0: 0.0 (0)
			MES 1: 9.5 (8)
			MES 2: 52.4 (44)
			MES 3: 38.1 (32)
**Biologic-naive**	59.5 (97)	64.9 (48)	55.1 (49)
**Therapy received at baseline**			
** Aminosalicylates**	46.0 (75)	16.2 (12)	70.8 (63)
** Steroids**	41.4 (67)	32.4 (24)	48.3 (43)
** Immunosuppressants (AZA/6-MP, MTX)**	15.3 (25)	14.9 (11)	15.7 (14)
** Anti-TNF**	55.2 (90)	71.6 (53)	41.6 (37)
** Anti-integrin**	20.9 (34)	12.2 (9)	28.1 (25)
** Anti-IL-12/23**	16.6 (27)	16.2 (12)	16.9 (15)
** JAK inhibitor (tofacitinib)**	8.0 (13)	1.4 (1)	13.5 (12)
** Others**	1.2 (2)	0 (0)	2.2 (2)
**Previous biologic therapy**			
** Anti-TNF**	52.6 (51)	45.9 (22)	59.2 (29)
** Anti-integrin**	13.4 (13)	6.3 (3)	20.4 (10)
** Anti-IL-12/23**	1.0 (1)	2.1 (1)	0.0 (0)
** JAK inhibitor (tofacitinib)**	1.0 (1)	0.0 (0)	2.1 (1)

Values are median (interquartile range) or % (n).

Abbreviations: AZA, azathioprine; CD, Crohn’s disease; CRP, C-reactive protein; FCAL, fecal calprotectin; GI, gastrointestinal; HBI, Harvey-Bradshaw Index; IBD, inflammatory bowel disease; IL, interleukin; ITT, intention to treat; JAK, Janus kinase; MTX, methotrexate; SCCAI, Simple Clinical Colitis Activity Index; SES-CD, Simple Endoscopic Score for Crohn’s Disease; TNF, tumor necrosis factor; UC, ulcerative colitis; 6-MP, 6-mercaptopurine.

**Table 2:** Clinical, biochemical, sonographic, and endoscopic cross-sectional results for week 12 and week 52.

	**IBD (n** = **163)**	**CD (n** = **74)**	**UC (n** = **89)**
**Result at week 12**			
**Transmural response**	60.7 (99)	55.4 (41)	65.2 (58)
**Transmural healing**	20.2 (33)	16.2 (12)	23.6 (21)
**Clinical response**	87.1 (142)	90.5 (67)	84.3 (75)
**Clinical remission**	68.1 (111)	78.4 (58)	59.6 (53)
**Clinical remission and transmural response**	44.2 (72)	44.6 (33)	43.8 (39)
**Clinical remission and transmural healing**	16.0 (26)	12.2 (9)	19.1 (17)
**Biochemical remission**	16.0 (26)	16.2 (12)	15.7 (14)
**Steroid-free remission**	36.8 (60)	50.0 (37)	25.8 (23)
**Endoscopic remission**	9.2 (15)	2.7 (2)	14.6 (13)
**Result at week 52**			
**Transmural response**	72.4 (118)	66.2 (49)	77.5 (69)
**Transmural healing**	33.7 (55)	27.0 (20)	39.3 (35)
**Clinical response**	90.2 (147)	90.5 (67)	89.9 (80)
**Clinical remission**	76.1 (124)	82.4 (61)	70.8 (63)
**Clinical remission and transmural response**	58.9 (96)	56.8 (42)	60.7 (54)
**Clinical remission and transmural healing**	28.8 (47)	23.0 (17)	33.7 (30)
**Biochemical remission**	11.7 (19)	13.5 (10)	10.1 (9)
**Steroid-free remission**	65.6 (107)	73.0 (54)	59.6 (53)
**Endoscopic remission**	17.2 (28)	6.8 (5)	25.8 (23)
**Number of flares after week 12**			
**0**	72.4 (118)	79.7 (59)	66.3 (59)
**1**	14.1 (23)	14.9 (11)	13.5 (12)
**2**	10.4 (17)	4.1 (3)	15.7 (14)
**3**	3.1 (5)	1.4 (1)	4.5 (4)
**IBD-related hospitalization or surgery**	9.8 (16)	13.5 (10)	6.7 (6)

Values are % (n).

Abbreviations: CD, Crohn’s disease; IBD, inflammatory bowel disease; UC, ulcerative colitis.

Statistical analyses were performed using SPSS version 26.0 (IBM).

### Ethical approval

The protocol was approved by the ethics committee of the Aerztekammer Niedersachsen, Germany (Bo/30/34/2018) on December 14, 2018. The study was conducted in accordance with the principles of the Declaration of Helsinki.

## Results

### Patient demographics

In total, 282 patients with IBD with active disease (HBI ≥ 7 or SCCAI ≥ 5) and increased BWT at baseline (>4 mm for sigmoid colon, >3 mm for other colonic segments and terminal ileum) were enrolled. Of these, 163 patients with IBD had a documented visit after 12 and 52 weeks and are thus presented as the intention-to-treat (ITT) population in this analysis. Of the ITT population, 45.4% (n = 74) were diagnosed with CD, while 54.6% (n = 89) were patients with UC. Patients had a median age of 34.6 years (IQR: 27.8-51.9 years) and a median disease duration at the time of study inclusion of 5.21 years (IQR: 1.74-10.69 years). As required by the inclusion criteria, patients were in clinical flare at baseline, as depicted by a median HBI of 10.0 (IQR: 8.0-12.0) in patients with CD and a median SCCAI of 8.0 (IQR: 7.0-10.0) in patients with UC. Laboratory parameters for CRP and FCAL were elevated as well, and patients showed endoscopic inflammation at the time of their last endoscopy prior to study start (CD: SES-CD 11.00 [IQR: 8.00-16.00], n = 29; UC: 85.4% [n = 76] with a Mayo endoscopic subscore of 2 or 3). Based on the last endoscopic assessment, patients with CD predominantly exhibited ileal-colonic (37.8% [n = 28]) or ileal (31.1% [n = 23]) disease extension. Patients with UC primarily had a pancolitis (41.6% [n = 37]). More than half of all patients (59.5% [n = 97]) were biologic-naive at baseline. Biologic-experienced patients had been mainly exposed to anti-TNF therapy (52.6% [n = 51]). This analysis population reflects a typical cohort of patients with moderate-severe CD or UC at the time of study conduction. Detailed patient demographics can be found in Table 1.

### IUS analysis at baseline

**Figure 1:**
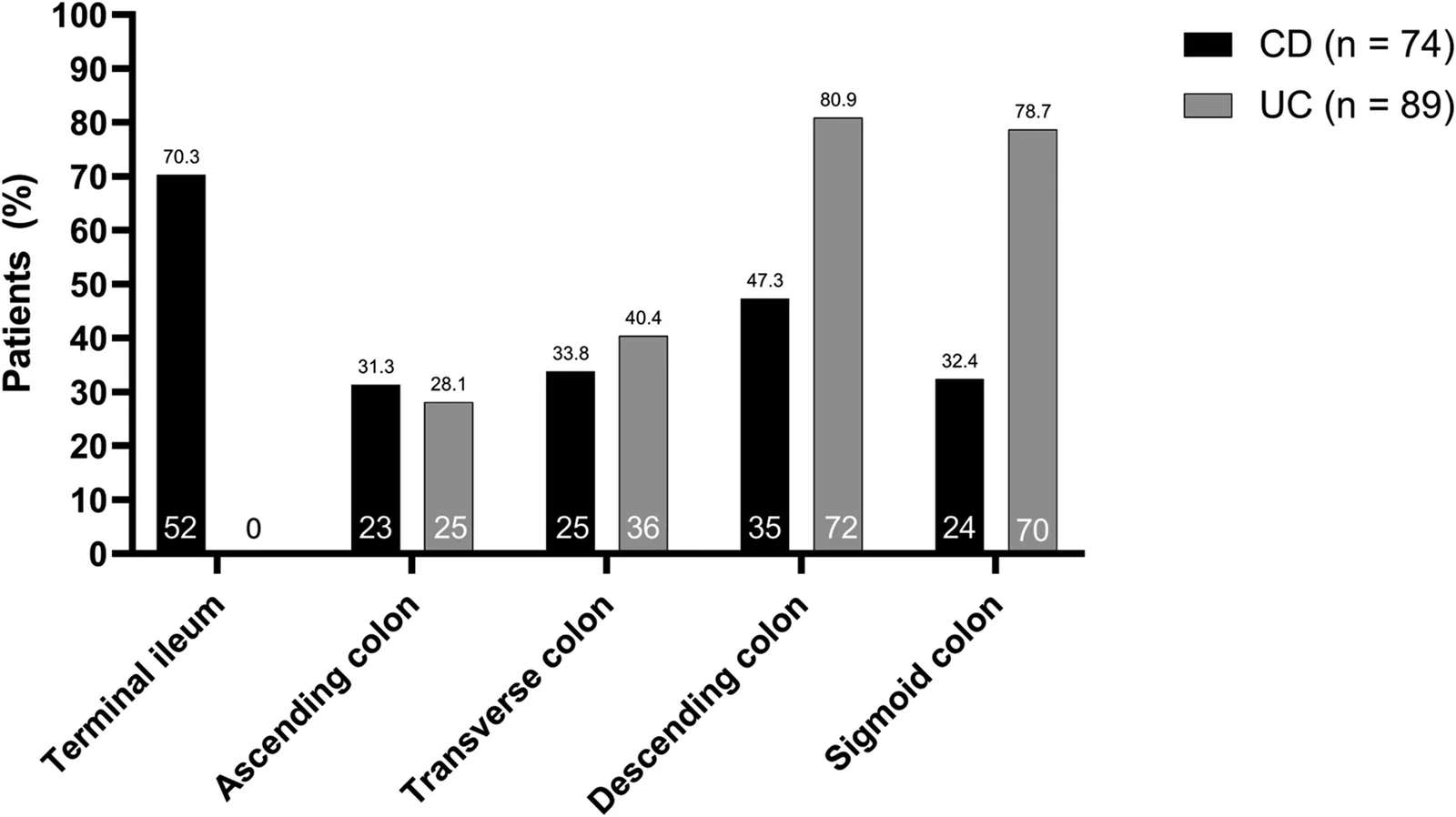
Proportion of patients with most affected segment at baseline in Crohn’s disease (CD) (black) and ulcerative colitis (UC) (gray) patients. Most affected was defined as >4 mm for the sigmoid colon and >3 mm for other colonic segments and terminal ileum (only assessed in CD).

At baseline, the IUS assessment revealed that the segments affected most frequently were the terminal ileum in patients with CD (70.3% [n = 52]) and the descending colon in patients with UC (80.9% [n = 72]), closely followed by the sigmoid colon (78.7% [n = 70]) (see Figure 1). The most affected bowel segment, defined by the greatest wall thickening, was the terminal ileum in patients with CD (44.6% [n = 33]) and the sigmoid colon in patients with UC (67.4% [n = 60]).

The most affected segment remained the same segment over the study course (data not shown). Median BWT at baseline was 4.50 mm (IQR: 3.15-6.30 mm) for the terminal ileum in patients with CD and 5.40 mm (IQR: 4.15-6.20 mm) for the sigmoid colon in patients with UC. In addition to an increased BWT at baseline, for 37.8% (n = 28) of patients with CD and for 49.4% (n = 44) of patients with UC an increased CDS signal (corresponding to Limberg 3 and 4 in CD) was documented. Further IUS pathologies were common in both IBD entities, as reflected by almost half of all patients with IBD (n = 68) having a loss of bowel wall stratification and every third IBD patient presenting with inflammatory fat (38.0% [n = 62]) and loss of haustration (31.3% [n = 51]). Of note, patients with CD more frequently had inflammatory fat than patients with UC (45.9% vs 31.5%). A thorough characterization of patients by IUS assessment is displayed in Table 2 and Table S1.

### Clinical disease activity over the study period

In the ITT population, the rate of clinical response of 87.1% (n = 142) at week 12 further increased to 90.2% (n = 147) at week 52. In agreement with the results for clinical response, 68.1% (n = 111) and 76.1% (n = 124) of patients with IBD were in clinical remission at week 12 and week 52, respectively. In total, 65.6% (n = 107) of patients with IBD were in clinical remission and steroid-free at week 52. A similar pattern was observed when patients with CD and patients with UC were evaluated separately. Patients with CD tended to achieve slightly higher rates of clinical response (week 12: CD, 90.5% [n = 67]; UC, 84.3% [n = 75]; week 52: CD, 90.5% [n = 67]; UC: 89.9% [n = 80]) and clinical remission (week 12: CD, 78.4% [n = 58]; UC, 59.6% [n = 53]; week 52: CD, 82.4% [n = 61]; UC, 70.8% [n = 63]) at week 12 and week 52. Mean and SD for clinical disease activity assessed by the HBI for patients with CD and SCCAI for patients with UC are depicted in Figure S3. Most patients remained flare-free after week 12 (72.4% [n = 118]). Endoscopic data were available only for a minority of patients and were more frequently available in patients with UC vs patients with CD. This may be attributed to the fact that sigmoidoscopy was more commonly applied in patients with UC than complete colonoscopy in patients with CD. At week 52, 6.8% (n = 5) of patients with CD and 25.8% (n = 23) of patients with UC achieved endoscopic remission. Details on the clinical status of patients at week 12 and week 52 can be found in Table 2.

### Biochemical activity over the study period and correlation to BWT

**Figure 2:**
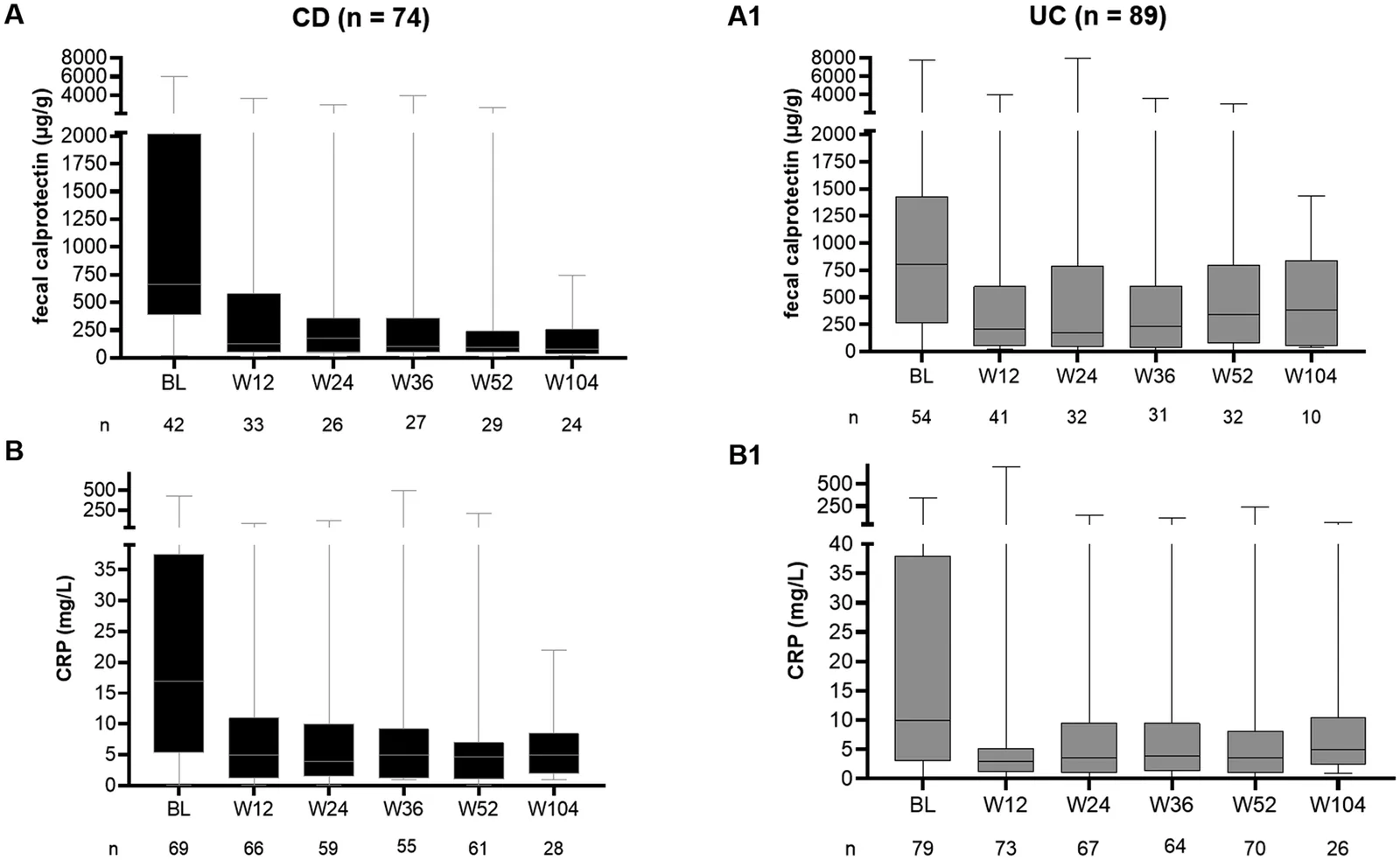
Median (A, A1) fecal calprotectin and (B, B1) C-reactive protein (CRP) levels over the study period for (A, B) patients with Crohn’s disease (CD) and (A1, B1) patients with ulcerative colitis (UC). BL, baseline; W, week.

In addition to clinical data, biochemical parameters for FCAL and CRP were assessed (Figure 2).

In patients with CD, median FCAL levels were reduced from 665 µg/g (IQR: 386-2024 µg/g) at baseline to 129 µg/g (IQR: 50-579 µg/g) after 12 weeks. Levels remained low until week 52 with 90 µg/g (IQR: 50-240 µg/g). FCAL levels were even higher at baseline in patients with UC vs patients with CD. A FCAL reduction from 800 µg/g (IQR: 264-1427 µg/g) at baseline to 205 µg/g (IQR: 51-600 µg/g) at week 12 and to 339 µg/g (IQR: 75-801 µg/g) was documented. At week 12 and week 52, 57.6% (n = 19 of 33) and 75.9% (n = 22 of 29) of patients with CD had achieved FCAL <250 µg/g, which was only the case for 56.1% (n = 23 of 41) of patients with UC at week 12 and 43.8% (n = 14 of 32) at week 52. Thus, in patients with UC, a similar trend in FCAL normalization below 250 µg/g was observed for the first 12 weeks, as described for patients with CD, but the proportion of patients with UC achieving FCAL normalization was diminished after week 12 until week 52 (Figure S1).

At baseline, patients with CD had higher CRP levels than patients with UC (median CRP 16.9 mg/L [IQR: 5.3-37.50 mg/L] vs 10.0 mg/L [IQR: 3.0-38.0 mg/L]). This difference was maintained at week 12 and week 52, but CRP reductions were apparent for both entities (week 12: CD 5.0 mg/L [IQR: 1.2-11.0 mg/L], UC 3.0 mg/L [IQR: 1.2-5.2 mg/L]; week 52: CD 4.6 mg/L [IQR: 1.0-7.0 mg/L], UC: 3.55 mg/L [IQR: 1.0-8.1 mg/L]).

In a post hoc analysis, we examined the correlation between changes in IUS parameters (BWT) and levels of FCAL and CRP throughout the study. At baseline and at 12 months, we observed a moderate correlation between BWT and FCAL for the sigmoid colon in UC (Spearman r = 0.341 and r = 0.623, respectively), while correlations with CRP were weaker (Tables S2 and S3).

### IUS parameters and transmural activity over the study period

**Figure 3:**
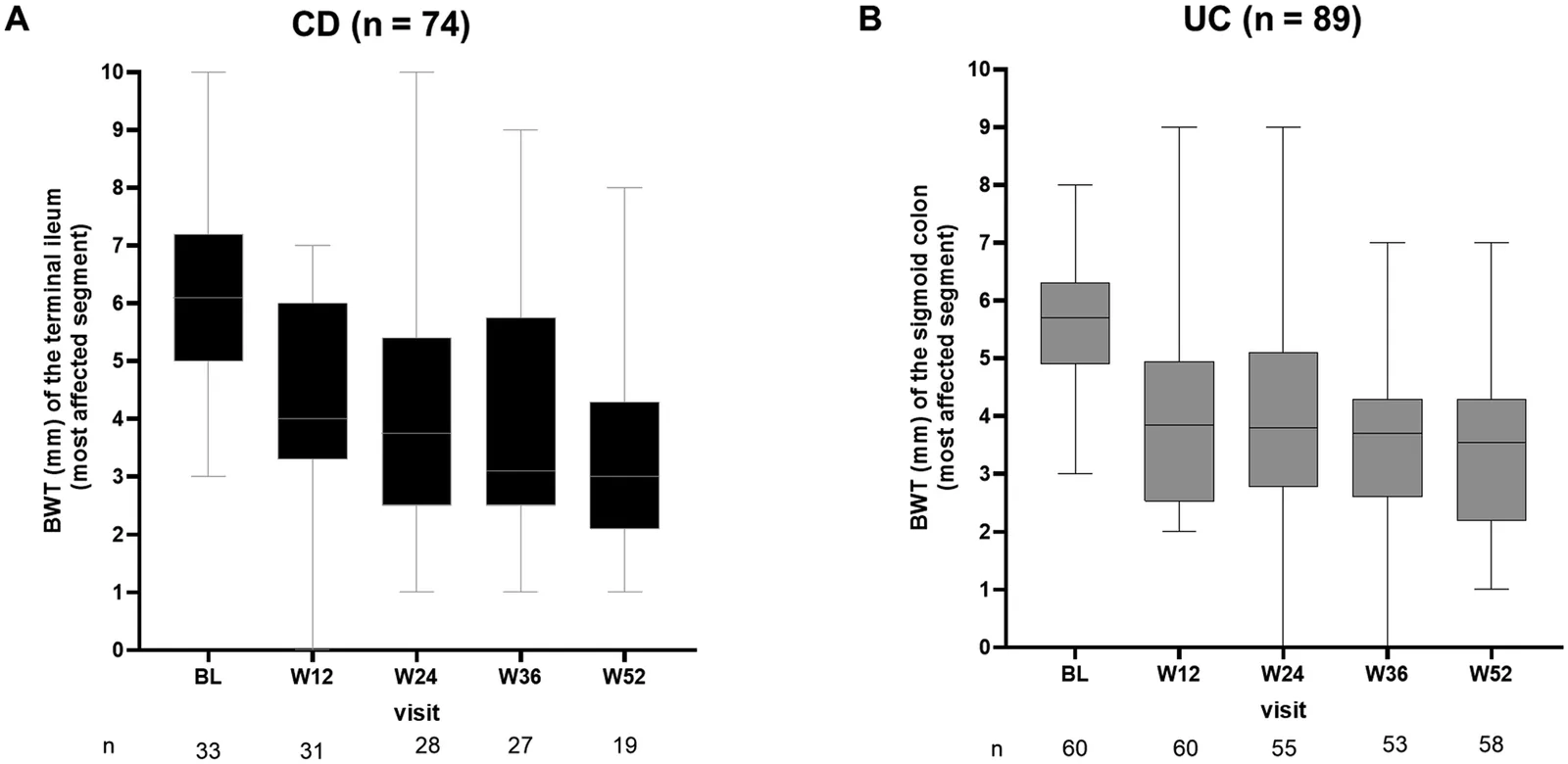
Bowel wall thickness (BWT) (most thickened segment) of the terminal ileum in patients with (A) Crohn’s disease (CD) and for the sigmoid colon in (B) patients with ulcerative colitis (UC) over the study period. BL, baseline; W, week.

Alongside clinical and biochemical improvements, patients with IBD showed substantial reductions in BWT as early as 12 weeks after therapy initiation, which were maintained throughout the study period. In patients with CD for whom the terminal ileum was deemed the most affected segment, the median BWT was reduced from 6.1 mm (IQR: 5.0-7.2 mm) at baseline, to 4.0 mm (IQR: 3.3-6.0 mm) at week 12 and to 3.0 mm (IQR: 2.1-4.3 mm) at week 52. In patients with UC, the median BWT of the sigmoid colon (if most affected segment) was reduced from 5.7 mm (IQR: 4.9-6.3 mm) at baseline to 3.85 mm (IQR: 2.53-4.95 mm) at week 12 and to 3.55 mm (IQR: 2.2-4.3 mm) at week 52. Overall, BWT reductions were obvious in both patients with CD and UC, although the dynamics seemed to differ to some degree (Figure 3). While the BWT of the terminal ileum in patients with CD showed further reductions until week 52, BWT reduction of the sigmoid colon in patients with UC tended to reach a plateau at week 12. This was also the case when patients were stratified for BWT in terminal ileum vs any colonic segment instead of stratification for disease entity.

**Figure 4:**
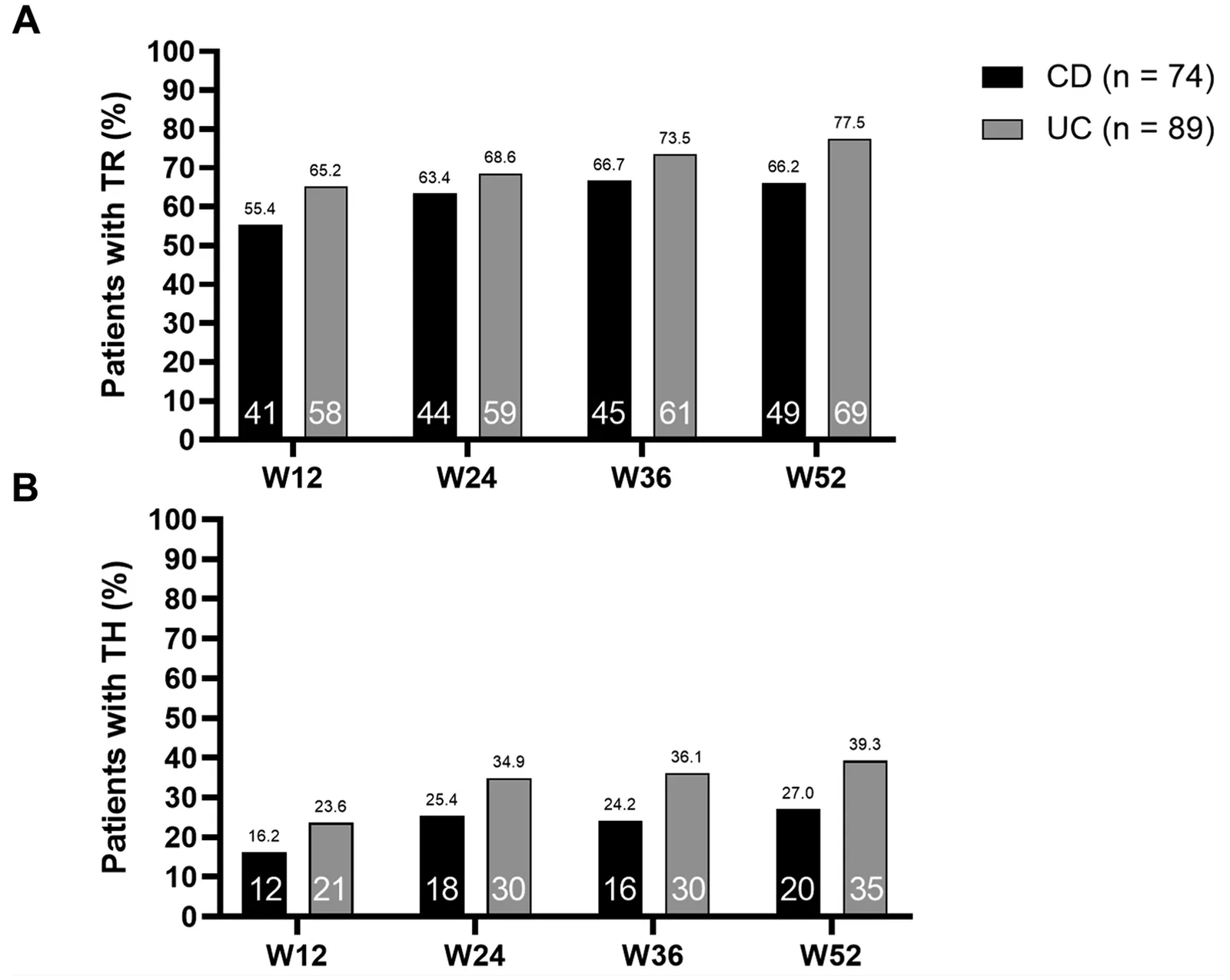
Rates of (A) transmural response (TR) and (B) transmural healing (TH) over the study course in Crohn’s disease (CD) (black) and ulcerative colitis (UC) (gray) patients. Patient numbers for week 12 (W12) to W52 were 74, 71, 66, and 74 for CD and 89, 89, 86, 83, and 89 for UC, respectively. Results based on nonresponder imputation analysis.

In the ITT, rates of transmural response and healing increased progressively from 60.7% (n = 99) at week 12 to 72.4% (n = 118) at week 52 (Figure 4A). Patients with UC depicted higher rates for both transmural endpoints over the study period. Transmural response at week 52 was sustained in 81.8% (n = 81) of patients with IBD with transmural response at week 12. Transmural healing, combining BWT and CDS, was not achieved as often as transmural response. Rates of transmural healing in all patients with IBD increased from 20.2% (n = 33) at week 12 to 33.7% (n = 55) at week 52 and were more pronounced in patients with UC than in patients with CD (Figure 4B).

The proportion of patients with additional IUS pathologies decreased over the study course, demonstrating a similar trend as BWT with the strongest amelioration observed at week 12 vs baseline (Figure S2).

### Evaluation of predictive value of transmural response

To evaluate early transmural response as a potential predictor of the outcome after 1 year, patients of the ITT were analyzed without stratification for CD vs UC to keep sufficient sample sizes.

**Figure 5:**
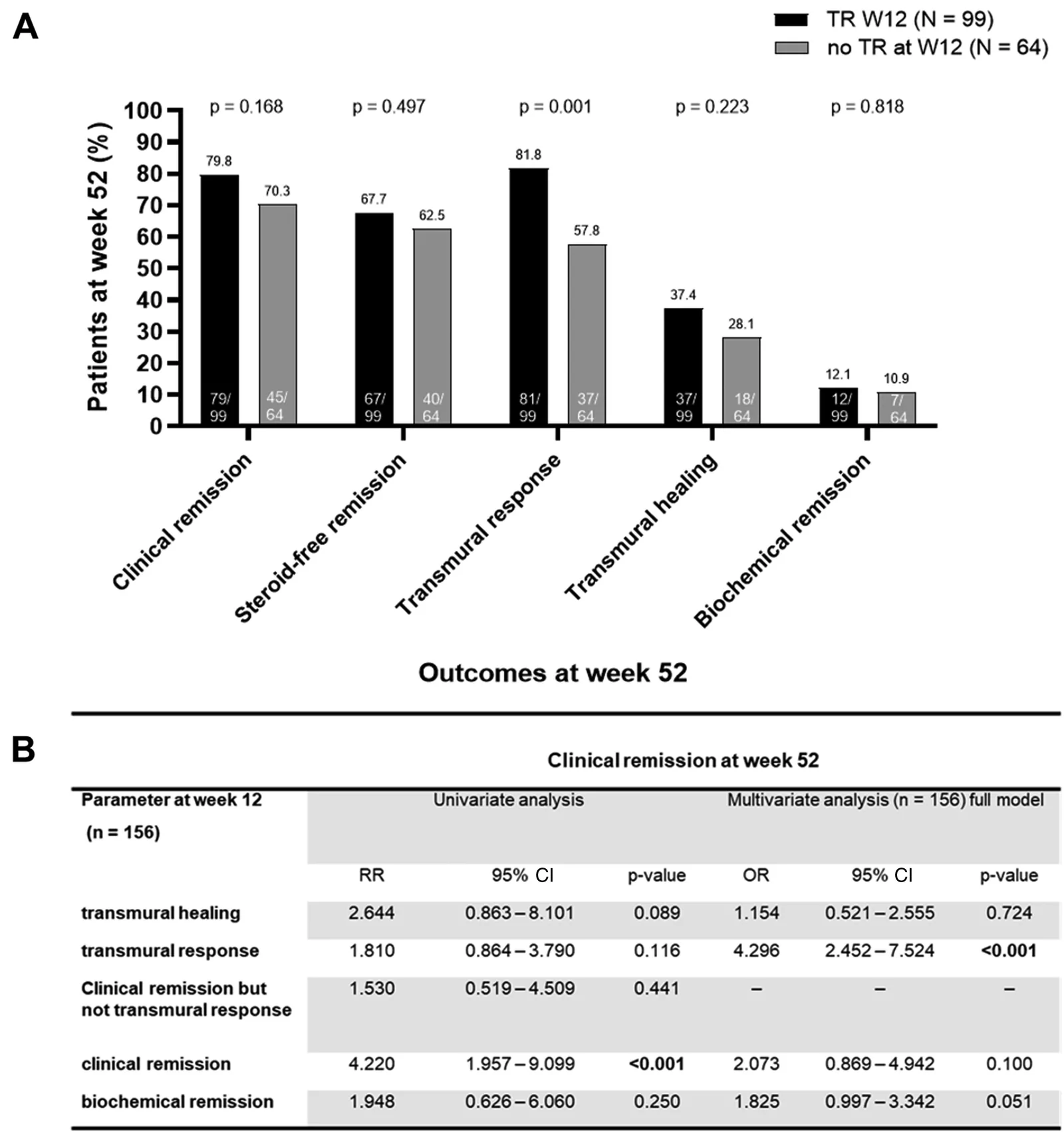
(A) Patients with inflammatory bowel disease (IBD) achieving week 52 (W52) outcomes (described on the x-axis) stratified for transmural response (TR) (yes, black vs no, gray), Mantel-Haenszel chi-square test, and (B) uni- and multivariate regression analysis to estimate the predictive value of clinical, biochemical, and sonographic parameters for clinical remission at week 52, results based on nonresponder imputation analysis. CI, confidence interval; OR, odds ratio; RR, relative risk.

A numerically greater proportion of patients with transmural response vs those without transmural response at week 12 achieved the evaluated outcomes at week 52 (Figure 5). Here, deltas of almost 10% were observed for clinical remission: approximately 5% for steroid-free remission, 24% for transmural response, approximately 10% for transmural healing, and approximately 2% for biochemical remission, all analyzed at week 52. The only statistically significant difference was found for transmural response at week 52.

Patients with IBD with transmural response at week 12 were numerically more likely to be without flares and complication (hospitalization or surgery) until week 52 compared with patients without transmural response at week 12 (with transmural response: 69.7% [n = 69 of 99] flare/complication-free; without transmural response: 57.8% [n = 37 or 64] flare/complication-free).

To estimate the predictive value of early improvements for the outcome at week 52, both a univariate and multivariate regression analysis were performed. On univariate analysis, clinical remission was the only statistically significant parameter. However, in a multivariate model, correcting for collinearity between the predictive variables, only a transmural response predicted clinical remission with statistical significance. Based on the full multivariate model, patients with a transmural response at week 12 are 4.3 times more likely to be in clinical remission at week 52.

## Discussion

The TRUST BEYOND study is a multicenter, prospective, noninterventional study that aimed to assess the predictive value of early transmural response assessed by IUS at week 12 for the outcome after 1 year in patients with IBD treated with standard of care medication in routine care.

The study demonstrated that pronounced rates of transmural response, which was defined as at least 25% reduction of BWT and/or normalization of BWT (≤3 mm) in the most affected segment (segment with the highest BWT) assessed by IUS, can be achieved in patients with both CD and UC after 3 months following treatment induction. Over the study course, rates of transmural response increased from 55.4% and 65.2% in patients with CD and UC at week 12 to 66.2% and 77.5% in patients with CD and UC, respectively. In the combined cohort, early transmural response was associated with clinical remission and reduced complications after 1 year of treatment.

“Overreliance” on clinical symptoms alone for managing IBD can lead to the risk of either overtreatment or undertreatment. With the advent of more treatment options and the capability to optimize therapy, IBD patient management is evolving toward a more personalized approach. This shift necessitates the use of reliable monitoring tools to support a T2T strategy to ultimately avoid disability and reduced quality of life. In this regard, several studies have evaluated the usage of IUS as a patient-centered, noninvasive, accurate approach in IBD patient management but often focused on patients with CD only.^5^^,^^27^

In this study, involving more than 40 IBD centers, we were able to show that in patients with both CD and UC BWT was reduced in line with clinical and biochemical improvements. In line with previous studies, the dynamics of transmural changes differed slightly between patients with CD and patients with UC. While in both patient cohorts, a marked increase in transmural response rates happened between baseline and week 12, and transmural response rates were higher in patients with UC than in patients with CD. Interestingly, median BWT was further reduced between week 12 until week 52 in patients with CD but not in patients with UC. This is in line with results of the TRUST&UC study, in which improvements were found as early as 2 weeks after treatment initiation. Differences in transmural response rates between the ileum and the colon have been previously demonstrated.^21^ Fibrosis and fibrostenosis could account for the differences in response rates with our IUS findings, further strengthening the concept of distinct pathophysiological mechanisms in ileal vs colonic inflammation as seen in patients with both CD and UC.^31^

A strength of this study lies in its prospective, longitudinal study design with reduced sampling bias because study sites were well balanced between clinical and office-based centers in Germany, Austria, and Switzerland. The study enabled close follow-up of patients, and in addition to IUS, clinical and laboratory parameters were documented as thoroughly as possible. An association between improvements in clinical and laboratory parameters and IUS findings was demonstrated, consistent with previous analyses. Recently, de Voogd et al^17^ demonstrated in a cohort of 40 patients with CD that a reduction in BWT of 4 to 8 weeks after anti-TNF initiation predicted endoscopic remission with high accuracy. For UC, Allocca et al^33^ recently found that the IUS improvement at week 12 was the only independent predictor of a Mayo endoscopic subscore ≤1 at the point of reassessment in a cohort of 49 patients with UC. In another study with 98 patients with UC, Milan ultrasound criteria (incorporating BWT and CDS) ≤6.2 at baseline was statistically significantly associated with a more favorable disease outcome after a median follow-up of 1.6 years.^34^ For new-onset patients with IBD, Madsen et al^34^ recently presented preliminary data indicating that baseline IUS activity can predict the need for biological therapy, IBD-related hospitalization, and surgery. Our study demonstrated the predictive value of IUS outcomes and thereby adds important value to these results given its multicenter, prospective nature.

While BWT, FCAL, and CRP all improved following treatment, the individual trajectories and the degree of correlation varied. This highlights the fact that changes in FCAL or CRP may not always mirror the degree of improvement seen with IUS. This pattern suggests that improvements in BWT generally parallel declines in biomarkers, but not perfectly so, and suggests the added value of multimodal monitoring.

A limitation of this study certainly lies in the limited number of documented endoscopies. As this was a noninterventional, real-world study, endoscopies were not systematically scheduled at fixed time points, but rather were performed at the discretion of the treating physician. As a result, endoscopic data at week 52 was available for only a minority of CD patients, and many procedures may have been performed outside the protocol window. This reflects routine clinical practice, but it also leads to lower reported remission rates, as less symptomatic or stable patients may not have received follow-up endoscopy. On the flip side, the relatively limited number of endoscopies performed may also serve as a reiteration of the physician-perceived strength (and confidence derived through the performance) of IUS in the therapeutic management of patients with IBD.

Effects of the COVID-19 pandemic cannot be ruled out and might have led to reductions in sample size. In addition, any changes to medication were determined by the investigators, resulting in small and diverse patient populations. Given that the study aimed to assess the feasibility and benefit of using IUS as a monitoring tool and its potential to predict disease outcomes in IBD, a detailed account of the medication changes was not provided. A higher proportion of biologic-naive patients in our cohort may have contributed to the pronounced IUS and clinical responses observed, as biologic-naive patients are known to respond more robustly to advanced therapies compared with biologic-experienced patients. We did not perform formal subgroup analyses by prior biologic exposure and thus cannot exclude the possibility that prior treatment history influenced the observed response rates. This should be considered when interpreting the results. More studies examining individual therapy responses are needed to clarify how specific medications affect IUS outcomes. This also applies to recently approved advanced therapies such as IL-23 antibodies or selective JAK inhibitors, which have not yet been sufficiently taken into account in this study. Systematic medication tracking will help us fully understand how consistent treatments impacts response dynamics.

It could also be noted that central reading of IUS results was not conducted, and interrater reliability was not assessed.

IUS is often criticized for its potential investigator dependency, an attribute inherent to all human-conducted diagnostic modalities. However, the most commonly used IUS parameters—BWT and CDS, as applied in this study—have demonstrated excellent interobserver variability in several independent evaluations. Taken together, our results highlight the utility of early IUS findings as potential predictors of long-term therapeutic response in patients with both CD and UC, providing a compelling argument for incorporating IUS in routine IBD management to guide treatment strategies effectively. Future studies should explore drug-dependent IUS findings and validate transmural response definitions in both patients with CD and UC.

## Supplementary Material

izag074_Supplementary_Data

## Data Availability

AbbVie is committed to responsible data sharing regarding the clinical trials we sponsor. This includes access to anonymized, individual, and trial-level data (analysis datasets), as well as other information (eg, protocols, clinical study reports, or analysis plans), as long as the trials are not part of an ongoing or planned regulatory submission. This includes requests for clinical trial data for unlicensed products and indications. These clinical trial data can be requested by any qualified researchers who engage in rigorous, independent, scientific research, and will be provided following review and approval of a research proposal, Statistical Analysis Plan, and execution of a Data Sharing Agreement. Data requests can be submitted at any time after approval in the United States and Europe and after acceptance of this manuscript for publication. The data will be accessible for 12 months, with possible extensions considered. For more information on the process or to submit a request, visit https://vivli.org/ourmember/abbvie/ then select “Home.”
