# Supplementary material for: Early transmural response assessed by intestinal ultrasound predicts long-term clinical remission in patients with IBD: Results from the TRUST BEYOND study
Source: Inflamm Bowel Dis. 2026 May 19;32(9):1770–82. doi: 10.1093/ibd/izag074 (PMC13533205; doi:10.1093/ibd/izag074)
Supplement: Supplementary file 1 [file izag074_supplementary_data.docx]

**Supplement**

**Supplemental Table T1: IUS characterisation of patients at baseline**

|  | | **IBD (n = 163)** | | **CD (n = 74)** | | | **UC (n = 89)** | | |
| --- | --- | --- | --- | --- | --- | --- | --- | --- | --- |
| BWT (mm, median and IQR)   - Terminal ileum - Ascending colon - Transverse colon - Descending colon - Sigmoid colon | 4.50 (3.15-6.30) (n=68)  2.50 (1.90-4.05) (n=128)  3.00 (2.00-4.60) (n=131)  4.10 (2.90-5.80) (n=151)  4.50 (3.10-6.00) (n=155) | | 4.50 (3.15-6.30) (n=68)  2.55 (2.00-4.40) (n=62)  2.95 (2.00-4.65) (n=60)  3.70 (2.10-5.30) (n=63)  3.25 (2.50-4.50 (n=66) | | | n.a.  2.45 (1-80-4.00) (n=66)  3.25 (2.00-4.50) (n=71)  4.45 (3.25-6.00) (n=88)  5.40 (4.15-6.20) (n=89) | | |  |
| Most affected segment at baseline, % (n)   - Terminal ileum - Ascending colon - Transverse colon - Descending colon - Sigmoid colon | 20.2 (33)  12.2 (20)  6.1 (10)  16.6 (27)  44.8 (73) | | 44.6 (33)  16.2 (12)  8.1 (6)  13.5 (10)  17.6 (13) | | | 0.0 (0)  9.0 (8)  4.5 (4)  19.1 (17)  67.4 (60) | | |  |
| Colour Doppler signal, % (n) | See IBD subtype | | Limberg 1 – normal signal: 14.9 (11)  Limberg 2 – mild signal: 37.8 (28)  Limberg 3 – moderate signal: 29.7 (22)  Limberg 4 – severe signal: 8.1 (6)  Not assessable: 9.5 (7) | | | Increased signal:  49.4 (44) | | |  |
| Segment with most increased Colour Doppler signal, % (n)   - Terminal ileum - Ascending colon - Transverse colon - Descending colon - Sigmoid colon | 14.1 (23)  9.2 (15)  3.1 (5)  8.0 (13)  21.5 (35) | | 31.1 (23)  8.1 (6)  4.1 (3)  5.4 (4)  14.9 (11) | | | n.a.  10.1 (9)  2.2 (2)  10.1 (9)  27.0 (24) | | |  |
| IUS pathologies   - Loss of bowel wall stratification, % (n) - Loss of haustration, % (n) - Presence of inflammatory fat, % (n) - Presence of fistula, % (n) - Presence of abscesses, % (n) - Presence of stenoses, % (n) | 41.7 (68)  31.3 (51)  38.0 (62)  1.2 (2)  1.2 (2)  5.5 (9) | | 50.0 (37)  n.a.  45.9 (34)  2.7 (2)  2.7 (2)  12.2 (9) | | 34.8 (31)  57.3 (51)  31.5 (28)  n.a.  n.a.  n.a. | | |  |  |

Abbreviations: IUS: intestinal ultrasound; IBD: inflammatory bowel diseases; CD: Crohn’s disease; UC: ulcerative colitis; BWT: bowel wall thickness

**Supplemental Table T2: Correlation of BWT and calprotectin**

Correlation analyses were performed via Spearman correlation coefficient for groups. Only coefficient values based on n ≥ 30 are useful (within the tables printed in bold).

**Correlation of BWT at the most affected segment and Calprotectin at all visits**

| **BWT** | **Visit** | **All (n)** | **r_Spearman_** | **CD (n)** | **r_Spearman_** | **UC (n)** | **r_Spearman_** |
| --- | --- | --- | --- | --- | --- | --- | --- |
| ***Colon sigmoideum*** | | | | | | | |
|  | Baseline (T0) | **44** | **0.422** | 9 | **0.653** | **35** | **0.341** |
|  | 3 months (T1) | **31** | **0.264** | 5 | 0.500 | 26 | 0.184 |
|  | 6 months (T2) | 26 | 0.143 | 5 | -0.600 | 21 | 0.181 |
|  | 9 months (T3) | 22 | 0.462 | 2 | - | 20 | 0.370 |
|  | 12 months (T4) | 27 | 0.627 | 5 | 0.000 | 22 | 0.623 |
|  | 24 months (T5) | 10 | 0.571 | 1 | - | 9 | 0.417 |

**Supplemental Table T3: Correlation of BWT and CRP**

Correlation analyses were performed via Spearman correlation coefficient for groups. Only coefficient values based on n ≥ 30 are useful (within the tables printed in bold).

**Correlation of BWT at the most affected segment and CRP at all visits**

| **BWT** | **Visit** | **All (n)** | **r_Spearman_** | **CD (n)** | **r_Spearman_** | **UC (n)** | **r_Spearman_** |
| --- | --- | --- | --- | --- | --- | --- | --- |
| ***Colon sigmoideum*** | | | | | | | |
|  | Baseline (T0) | **63** | **0.222** | 12 | 0.179 | **51** | **0.236** |
|  | 3 months (T1) | **59** | **0.081** | 12 | -0.413 | **47** | **0.206** |
|  | 6 months (T2) | **54** | **0.210** | 10 | -0.018 | **44** | **0.260** |
|  | 9 months (T3) | **46** | **0.246** | 6 | 0.486 | **40** | **0.223** |
|  | 12 months (T4) | **54** | **0.271** | 7 | -0.036 | **47** | **0.295** |
|  | 24 months (T5) | 22 | -0.028 | 3 | -0.500 | 19 | -0.004 |

**Supplemental Figure S1: Faecal calprotectin normalisation below 250 µg/g in CD (black) and UC patients (grey), (n/N with N depicting number of patients with available data).**

Abbreviations: FCAL: faecal calprotectin; CD: Crohn’s disease; UC: ulcerative colitis; BL: baseline; W: week

**Supplemental figure S2: Proportions of CD patients (A) and UC patients (B) with disease-specific IUS pathologies over the study course. W – week, results based on non-responder imputation analysis.**

Abbreviations: CD: Crohn’s disease; UC: ulcerative colitis; BL: baseline; W: week; IUS: intestinal ultrasound

**Supplemental figure S3: Clinical disease activity over the study period defined by Harvey-Bradshaw-Index in CD patients (A) and Short Clinical Colitis Activity Index in UC patients (B).**

Abbreviations: CD: Crohn’s disease; UC: ulcerative colitis; HBI: Harvey-Bradshaw Index; SCCAI: Simple Clinical Colitis Activity Index; BL: baseline; W: week
